# Resource development in otolaryngology-head and neck surgery: an analysis on patient education resource development

**DOI:** 10.1186/s40463-014-0027-5

**Published:** 2014-07-16

**Authors:** Jeremy Goldfarb, Vishaal Gupta, Heather Sampson, Albino Chiodo

**Affiliations:** 1 Undergraduate Medical Education, Faculty of Medicine, University of Toronto, Toronto, Canada; 2 Department of Family and Community Medicine, University of Toronto, Office of Research, Toronto East General Hospital, 825 Coxwell Avenue, Toronto M4C 3E7, Ontario, Canada; 3 Department of Otolaryngology – Head and Neck Surgery, University of Toronto, Toronto East General Hospital, 825 Coxwell Avenue, Toronto M4C 3E7, Ontario, Canada

**Keywords:** Patient education, Resource development, Otolaryngology-head and neck surgery, Surgical complications, Post-operative period

## Abstract

**Background:**

There is a need for educational tools in the consenting process of otolaryngology-head and neck procedures. A development strategy for the creation of educational tools in otolaryngology-head and neck surgery, particularly pamphlets on the peri-operative period in an adenotonsillectomy, is described.

**Methods:**

A participatory design approach, which engages key stakeholders in the development of an educational tool, is used. Pamphlets were created through a review of traditional and grey literature and then reviewed by a community expert in the field. The pamphlets were then reviewed by an interdisciplinary team including educational experts, and finally by less vulnerable members of the target population. Questionnaires evaluating the pamphlets’ content, layout, style, and general qualitative features were included.

**Results:**

The pamphlets yielded high ratings across all domains regardless of patient population. General feedback was provided by a non-vulnerable patient population and final pamphlets were drafted.

**Conclusions:**

By using a participatory design model, the pamphlets are written at an appropriate educational level to incorporate a broad audience. Furthermore, this methodology can be used in future resource development of educational tools.

## Introduction

Currently 19 out of 10,000 Canadian children receive an adenotonsillectomy [[1]]. It is one of the most common procedures performed by otolaryngologists-head and neck surgeons, and is also one of the most common operations in the pediatric population. Although the procedure is so widely performed, the intra-operative and post-operative periods can be quite stressful, and caring for a child undergoing surgery can be very challenging for guardians, necessitating proper supports.

During the intra-operative and post-operative periods, it is particularly important that caregivers are provided with effective education. Previous studies have looked into the informed consent process, and the effect verbal and written consenting tools have on patient knowledge of these periods. Aremuu et al. [[2]] demonstrated that the addition of a handout significantly altered recall of potential complications in otolaryngology-head and neck surgeries. Furthermore, Le et al. [[3]] found that although most parents were satisfied with the preoperative counseling, 94% of patients felt that a postoperative phone call the day after surgery was helpful; despite counseling, they hadn’t realized how severe the throat pain would be. Kuo et al. [[4]] demonstrated that patients lack awareness of symptoms they might expect post-adenotonsillectomy. Throat pain, bleeding, and voice changes are all common during the post-operative period, and their triage requires an informed caregiver equipped with appropriate educational tools.

Even though there is an increasing need for educational tools in otolaryngology, the most effective medium for peri-operateive surgical education in adenotonsillectomy is still debated. Adams et al. [[5]] found no significant difference in knowledge retention between verbal counseling, counseling and a written handout, and counseling and a video, and also concluded that the otolaryngologist-head and neck surgeon remained the most important source of information. This study, however, was performed on well-educated caregivers and did not provide a pamphlet development procedure. A primer for surgical pamphlet development is currently missing in the literature.

The aim of this study is to provide an approach for developing educational tools in otolaryngology-head and neck surgery that will be effective across multiple populations.

## Methods

A mixed methods approach was utilized and adhered very closely to the methodology described by Adirim et al. [[6]] for developing and evaluating an educational pamphlet. Two pamphlets were drafted—an pre-operative pamphlet outlining complications and a post-operative pamphlet—describing the postoperative course or care of the patient.

### Development

The development portion was conducted using a participatory design approach, engaging key stakeholders [[7]].

#### Phase 1 – Pamphlet development and design

The pamphlets were created using a systematic review of the current informational landscape in adenotonsillectomy peri-operative care. A review of traditional literature was done and pertinent information was included in the pamphlets. Multiple search strategies in Ovid MEDLINE were utilized and four review papers were selective as information sources [[8]–[10]]. Additionally, an environmental scan including online grey literature and similar pamphlets available at other Canadian otolaryngology-head and neck surgery clinics was done. Pamphlets created by McGill University [[11]] and the University of Mississippi [[12]] were included in the review process. Grey literature was reviewed using popular search mediums available to patients. Utilizing multiple search strategies, google.com, yahoo.com, and bing.com were searched. Websites providing lay information on adenotonsillectomy were reviewed. The pamphlets were written to be inclusive of individuals with minimal educational background as per the recommendations of McAllister et al. [[13]]. All the information was reviewed to create draft pamphlets which were audited by an experienced community otolaryngologist-head and neck surgeon before proceeding to *Phase 2*.

#### Phase 2 – Critical evaluation by diverse healthcare professionals

The draft pamphlets from *Phase 1* were distributed to a team of healthcare professionals for critical evaluation of content and style. The pamphlets were given to two adenotonsillectomy-performing otolaryngologists-head and neck surgeons, three nurses, one speech language pathologist, two educational experts, and one plain text editor. The pamphlets were edited based on the qualitative feedback from this group. (see “Key Stakeholders”)

### Key stakeholders

 Otolaryngologist – head and neck surgeon

 Nurses in Otolaryngology

 Speech Language Pathologist

 Educational Experts

 Plain Text Editors

 Care-givers of Non-vulnerable Patients

 Fellow Medical Student

#### Phase 3 – Evaluation by less vulnerable members of the target population

This phase involved evaluation of style and content by guardians of children who have undergone adenotonsillectomy, and whose children have successfully recovered from the surgery. Guardians were contacted after the follow-up visit, once an otolaryngologist-head and neck surgeon ensured there were no post-operative complications. They were asked to read the pamphlets and fill out a brief questionnaire to elicit qualitative feedback (see ‘Patient Questionnaires’ subsection). The pamphlets were again modified based on the target audience’s evaluation, and final pamphlets were drafted. The final pamphlets were then assessed for readability using the Flesch-Kincaid readability test.

### Patient questionnaire

Adenotonsillectomy Qualitative Feedback Questionnaire:

Thank you for your agreeing to participate on our resource development project. We very much appreciate your time and valuable feedback.

Name:

Relation to patient:

Surgical Complications Pamphlet:

 Please provide feedback on the content of the pamphlet. (Was it too difficult? Is it appropriate for the target population? Should anything be removed? Should anything be added?)

 Please provide feedback on the style of the pamphlet (Was the syntax appropriate? Was the language confusing?)

 Please provide feedback on the general layout of the pamphlet (Were the sections appropriately titled? Were the colours acceptable? Were the visual images appropriate?)

 General Feedback:

Post-Operative CarePamphlet:

 Please provide feedback on the content of the pamphlet. (Was it too difficult? Is it appropriate for the target population? Should anything be removed? Should anything be added?)

 Please provide feedback on the style of the pamphlet (Was the syntax appropriate? Was the language confusing?)

 Please provide feedback on the general layout of the pamphlet (Were the sections appropriately titled? Were the colours acceptable? Were the visual images appropriate?)

**Figure 1:**
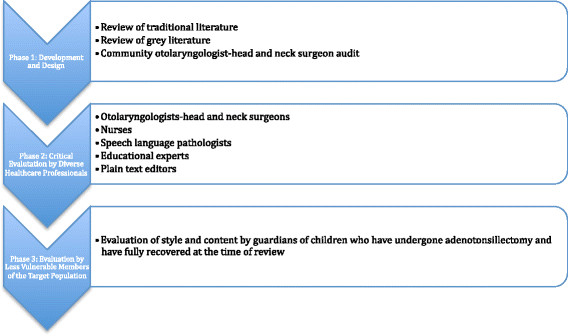
Development and evaluation workflow.

 General Feedback:

Figure 1 Development and evaluation process.

## Results

The pamphlets were developed using the methods outlined. A multidisciplinary team was engaged that provided feedback on content, style, appropriateness of language, and overall effectiveness. The pamphlets were edited five times in an iterative process based on the feedback provided. Educational experts provided feedback on language difficulty and recommended simplification. Other themes that emerged in the multidisciplinary review were improving content layout and providing additional information on appropriate patient triage. These changes were made and incorporated into the draft given to non-vulnerable patients.

**Figure 2:**
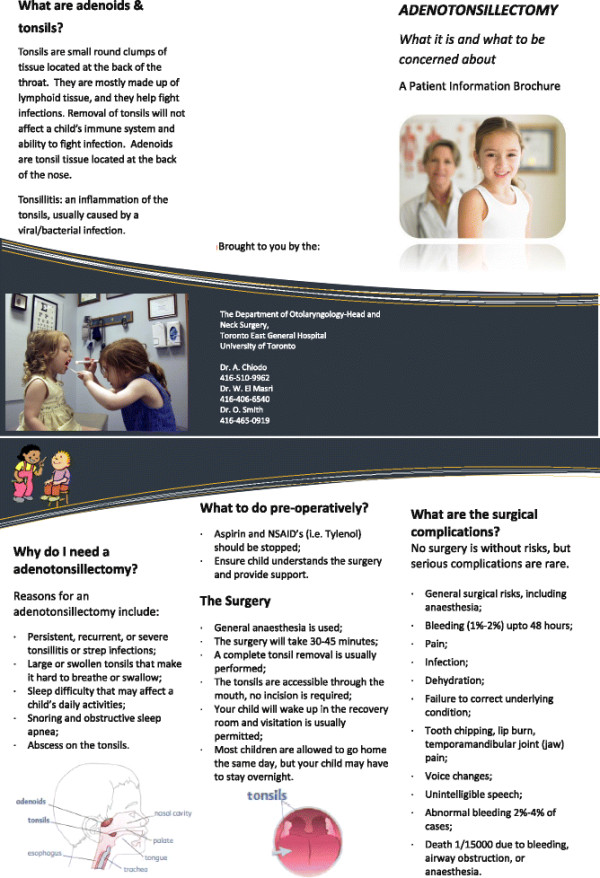
Surgical complications pamphlet.

**Figure 3:**
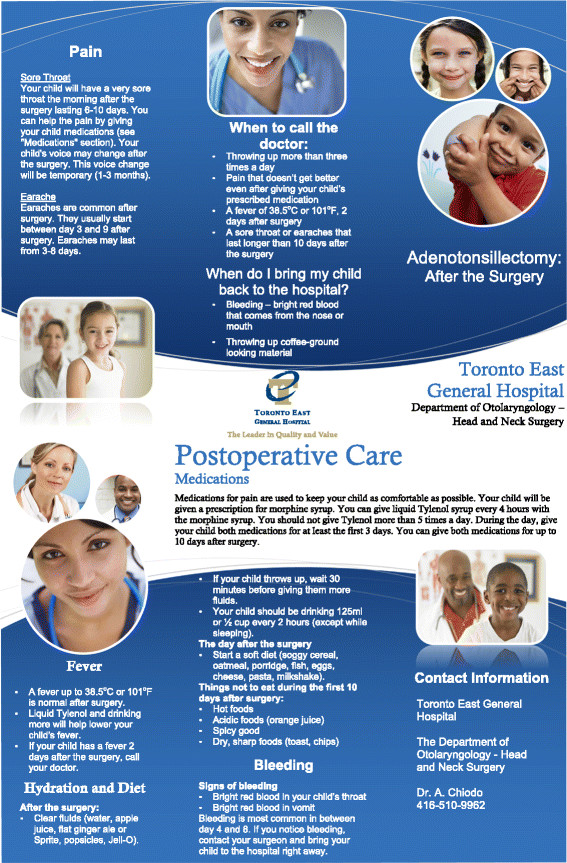
Post-operative care pamphlet.

Ten non-vulnerable patients were then approached to participate in *Part 1, Phase 3* of the study. Ten patient-guardians completed the questionnaire during their intra-operative and post-operative visit. Nine of the ten participants had positive or no feedback on content, style, or layout. (Table 1) These changes were made and final pamphlets (Figures 2 and 3) were designed. The final pamphlets were also scored on the Flesch-Kincaid readability test. The Surgical Complications pamphlet scored at a Grade 11.5 level and the Post-Operative Complications pamphlet at a Grade 8.3 level.

**Table 1:** Feedback from care-givers of non-vulnerable patients

**Domain:**	**Feedback (Quotes):**
**General:**	“Additional online resources should be provided”
	“How much bleeding requires as emergency room visit?”
**Content, Style, and Layout:**	“Very easy to follow”
	“Reflected my discussion with the doctor well”
	“Layout was a bit confusing”
	“Some language was too complicated”

## Discussion

Educational materials are of limited value if patients cannot understand their content [[4]]; therefore, this resource development study aimed to create and evaluate a peri-operative pamphlet for caregivers of children undergoing adenotonsillectomy to ensure it can be understood and effective for patients of all backgrounds.

Development of an effective resource depends on creating a resource written at an appropriate educational level [[13]]. A participatory design model, engaging key stakeholders in the design process, can be used to develop an appropriate resource [[6]]. Once an appropriate resource has been developed, it was shown by Aremu et. al. [[2]] that handouts improve recall in otolaryngology-head and neck procedures (62% vs 51%). Knowing this, a participatory design model should be employed to create peri-operative pamphlets for paediatric caregivers of various backgrounds.

Due to the time intensive nature of our participatory design model, engaging a large sample population was not possible Therefore, the sample may not have been representative of the breadth of adenotonsillectomy patients, including non-English speakers and patients with poor literacy. Also, while the non-vulnerable patient caregivers positively reviewed the pamphlets, a more quantitative methodology assessing demographic information would be required to ensure the pamphlets are effective across socioeconomic strata. Ideally, the pamphlets would be reviewed by those with dyslexia, English as a second language, the visually impaired, and those with poor health literacy skills, to name a few, Once analyzed by these groups a more broadly inclusive pamphlet would be finalized.

## Conclusions

The aim of this study was to present an approach for development of future educational tools in otolaryngology-head and neck surgery. It is important for physicians, as advocates, to provide up-to-date and understandable educational tools for patients. The above methodology has shown to be effective for creating a preliminary educational tool. It emphasizes interdisciplinary collaboration as well as the inclusion of end-users in the developmental process. However, further studies need to be conducted to determine the efficacy of such tools in various patient populations.

### Recommendations and considerations

Utilizing the on-line format of this journal we would like to engage our colleagues in investigating the utility and value of this tool via the web based access (http://www.journalotohns.com/). We welcome readers/clinicians to download the brochure and questionnaires to use in their clinical environments. We look forward to receiving reader experience and evaluation of the patient education tool: we would be pleased to share recommended changes and edits to the brochure.

## Consent

Written informed consent was obtained from the patient’s guardian/parent/next of kin for the publication of this report and any accompanying images. Every effort was made to use stock images in the brochures.

## Competing interests

The authors of this article have no competing interests to disclose and no conflicts of interest.

## Authors’ contribution

VG and JG participated in the design of the study and drafted the manuscript. AC conceived of the study, carried out the study, and helped draft the manuscript. HS helped with study design, with data collection, and with drafting the manuscript. All authors read and approved the final manuscript.
